# Portrayal of Domestic Violence Trajectories During the Perinatal Period

**DOI:** 10.1177/10778012211014564

**Published:** 2021-06-15

**Authors:** Sylvie Lévesque, Carole Boulebsol, Geneviève Lessard, Mylene Bigaouette, Mylene Fernet, Alena Valderrama

**Affiliations:** 1 Université du Québec à Montréal, Canada; 2 Université de Montréal, Québec, Canada; 3 Université Laval, Québec, Canada; 4 Federation des maisons d’hébergement pour femmes, Montréal, Québec, Canada; 5 CHU Sainte-Justine, Montréal, Québec, Canada

**Keywords:** domestic violence, gender-based violence, perinatal period, qualitative study

## Abstract

Domestic violence during the perinatal period (DVPP) refers to the various ways that women’s partners or ex-partners control and coerce them during pregnancy and the 2 years postpartum. From the descriptions of 17 women with firsthand experience of DVPP, this article reports on its manifestations and the associated contexts. The results reveal escalating violence, diverse forms of violence, and exacerbated consequences over the perinatal period. The contexts that pose additional challenges for the women include financial precariousness and the partner’s substance abuse, and to a lesser extent the residential situation.

## Background

Domestic violence (DV) is a recognized social and public health issue (Public Health Agency of Canada, 2016). It is defined as gender-based violence against women by an intimate partner, including a current or ex-husband, cohabitating partner, boyfriend, or lover (World Health Organization [WHO], 2013). Our conceptualization of DV is based on a feminist analysis that accounts for the historically inegalitarian social structures that impact women, and that recognizes that the consequences of DV are more serious for female than male victims (Hamberger & Larsen, 2015). Moreover, DV is not a homogeneous reality (Laing et al., 2013). Indeed, the trajectories of women victims of intimate partner violence (IPV) can be distinguished according to their age, sexual orientation, socioeconomic living conditions, ethnocultural origin, or migratory status, among other factors (Lessard et al., 2015). Thus, women do not constitute a homogeneous group. However, they represent the majority of victims of IPV, and men are the main perpetrators (WHO, 2013).

DV therefore takes different forms. However, they all involve an intimate partner who seeks to gain or maintain power over the other (Kelly & Johnson, 2008; Lessard et al., 2015). The different forms of DV—physical, sexual, psychological, verbal, economic, cybercontrol or cyberviolence, and spiritual—may be situated along a continuum that goes as far as femicide (Buchanan et al., 2014; Ford-Gilboe et al., 2016; Laforest, Maurice, & Bouchard, 2018). Moreover, DV can surface at any time in a woman’s life, including the perinatal period, which extends from the start of pregnancy up to the child’s second year of life.

### The Perinatal Period: Vulnerability During Pregnancy and the 2 Years Postpartum

The perinatal period is a time of vulnerability that brings changes to individual, conjugal, and family lives, and particularly when it involves a first child (Meleis, 2010; Van Parys et al., 2014). In Québec (Canada), a province-wide survey revealed that one mother in 10 with a child between 6 months and 5 years of age reported at least one episode of domestic violence during the perinatal period (DVPP) (Lévesque & Julien, 2019). These numbers are similar to those for all of Canada (Public Health Agency of Canada, 2009): 11% of Canadian women who were subjected to physical or sexual violence had experienced at least one violent episode during pregnancy. In most cases, the act of violence was committed by a partner, husband, or boyfriend. Furthermore, DVPP has negative consequences for the health and well-being of both the victimized women and their children (McMahon et al., 2011; Pastor-Moreno et al., 2020).

For some women, pregnancy acts as the starting point for the violence, whereas for others, violent behaviors that were ongoing increase or, on the contrary, decrease (Daoud et al., 2012; Taillieu & Brownridge, 2010). Whatever the form, these violent behaviors are neither isolated nor anecdotal, but instead recurrent (Taillieu & Brownridge, 2010). They may also be interspersed with calmer periods (Edin et al., 2010), as observed in DV situations outside of the perinatal period. To the various forms of DV during pregnancy, we may add the risk of reproductive coercion, which occurs when the partner uses force or threats to convince the woman to have an abortion (Edin et al., 2010), or in contrast, to continue an unwanted pregnancy (Lévesque et al., 2021). According to Samankasikorn et al. (2019), women who were subjected to DV before pregnancy have a nearly eightfold higher risk of reproductive coercion than women who were not. Furthermore, women who had experienced reproductive coercion in the form of birth control sabotage (e.g., destroying, hiding, or nonconsensual withdrawal of a condom) or physical violence were at higher risk of unwanted pregnancy than women who had not experienced these forms of violence (Samankasikorn et al., 2019). In this study, as in others, the postnatal period ranges from 0–2 years (Fogarty et al., 2018; Miller-Graff et al., 2019). More often than not, when children are not yet in school and are still very dependent, mothers remain primarily responsible for the care and education of their toddlers (Lessard et al., 2019). This is a period when men deploy new means of exercising violence against their spouse or ex-spouse by interfering with the exercise of their parenthood, in particular by attacking the parenting skills of mothers (Buchanan et al., 2014; Heward-Belle, 2017). The violence suffered by a mother when her child is very young can compromise the bonds of attachment, in addition to lowering the mother’s self-esteem (Buchanan et al., 2014).

### Contexts Associated With DVPP

The most predictive factor for DV during pregnancy appears to be previous violent behavior toward the woman in a conjugal, family, or other context. For instance, 60–96% of women who were victims of DV during pregnancy reported previous violent behavior in such contexts (Taillieu & Brownridge, 2010). Moreover, women who had experienced episodes of DV or family violence prior to getting pregnant were at fourfold higher risk of DV during pregnancy than women with no such history of violence (James et al., 2013). Another potential contributing factor for DV during pregnancy is separation from the violent partner, usually to protect the woman and the fetus (Brownridge et al., 2008). Studies on DVPP have identified many other risk factors, including individual, family, community, and social factors. Of these, we may highlight young age (generally, 20 years or less), low education level, precarious socioeconomic status, financial dependence on the partner, unmarried status, minority racial-ethnic identity, and partner’s alcohol and/or drug abuse (Bailey, 2010; Daoud et al., 2012; James et al., 2013; Taillieu & Brownridge, 2010). Furthermore, the socially constructed gender roles of different cultures can also influence the degree to which violence is accepted or tolerated (Taillieu & Brownridge, 2010).

The research describes a variety of ways that violence manifests during pregnancy, with disparities in the frequency, intensity, and severity of violent behaviors, depending on the countries, contexts, definitions, and measurement tools (James et al., 2013; Van Parys et al., 2014). However, few studies have addressed DVPP in the context of pregnancy combined with the first 2 years of the child’s life. In addition, most studies focus on one or a few forms of DV, which limits the consideration of interwoven forms. Accordingly, this article aims to (a) document how DV manifests over the entire perinatal period, and in all its recognized forms; and (b) identify the contexts that modulate DVPP and its consequences for the lives of mothers and their babies.

## Method

A qualitative exploration was conducted in collaboration with six co-researchers from different disciplines and universities as well as nine partners, including provincial community groups working with DV and perinatality. All the researchers and partners participated in the research orientation committee to develop the study design and recruitment strategy. As members of the committee responsible for the qualitative data drawn from the mothers’ testimonies, the co-authors actively contributed to design the data collection and analysis tools. The research strategy, based on the tenets of constructivist epistemology, was to engage women in conversations and encourage them to tell their stories (Creswell, 2013).

### Participants

The participants comprised 17 mothers living in the province of Québec (Canada) who were victims of DVPP. Table 1 shows that seven participants were born outside Canada. Three were 18–25 years old, seven were 26–35 years old, and seven were older than 36 years. Fourteen had one or two children, and two were pregnant at the time of the interview. Three quarters of the participants were no longer living as a couple with the perpetrator of the DVPP. Eleven had exclusive custody of their child or children, two were pursuing legal action to obtain custody, one no longer had custody, and one had shared custody. Twelve participants held a job, either full-time or part-time. All had experienced DVPP in the 5 years prior to the study.

**Table 1.** Participants’ Sociodemographic and Family Characteristics (*n* = 17).

Characteristics	*n*	%
Age (years)		
18–25	3	18
26–35	7	41
≥36	7	41
Country of birth		
Canada	10	59
Other	7	41
Sexual orientation		
Heterosexual	17	100
LGBTQ+	0	0
Education level		
Elementary/secondary	5	29
College (cégep)^a^	3	18
University	9	53
Occupation^b^		
Employed	12	70
Studying	3	18
At home	3	18
Seeking employment	2	12
Other	2	12
Relationship with the violent partner		
Separated	14	82
In a couple	3	18
Number of children		
One	8	47
Two	6	35
Three or more	3	18
Childcare arrangement		
Shared custody	1	6
Exclusive custody	11	64
No custody	1	6
Pursuing legal action to obtain exclusive custody	2	12
Living with the child’s father	2	12

*Note.* LGBTQ = lesbian, gay, bisexual, transgender, queer.

a Refers to a post-secondary program in Québec: either a pre-university or technical college stream.

b Because several participants are classified under more than one occupation, the total exceeds 100%.

### Procedure

This study obtained two ethical certifications: one from a university and another from a state institution to meet the demands of the health care network. To ensure recruitment feasibility at various institutional service points in that network, four more requests for ethical suitability were submitted and approved. All participants signed a consent form.

Participants were targeted according to three inclusion criteria: (a) aged 18 years or more at the time of the study (age of legal majority in Québec), (b) the biological mother of a child with whom she has had regular contact in the year prior to the study, and (c) had experienced DVPP in the last 5 years. Purposive sampling was used to recruit participants with diverse profiles (e.g., age, ethnocultural identity, place of residence, conjugal context, access to support). Three administrative regions of Québec were targeted to represent the realities of women living in urban as well as rural areas.

Several complementary recruitment strategies were used. A promotional video was sent to dozens of public institutions and community groups. Posters were circulated on websites and social networking sites and distributed to community groups working with DV and/or parents. Health care and social work professionals were asked to let mothers know about the study. Meetings were also held with managers and team leaders at health and social services centers.

Of the initial sample of 26 women who were willing to participate, nine did not meet the inclusion criteria and were not retained. Individual semi-directed interviews averaging 120 min each were held with the final sample (*n* = 17) from March 2018 to July 2019. Interviews were generally held at the university’s research facilities, and to a lesser extent, at sites chosen by the participants or via Zoom video conferencing. All participants received CAD$30 as compensation and received a list of specialized resources.

### Measures

Inspired by the precepts of the Life History Calendar of Domestic Violence (Yoshihama et al., 2002), a guide for the semi-structured interviews was developed to help the participants more accurately recall their DV experiences. Thus, by organizing their trajectories into themes over time, they could more easily recall their experiences and situations before, during, and after pregnancy until the child was 2 years old, in addition to describing their current situation. The main themes were the relational context, whether the pregnancy was planned or unplanned, the various manifestations of violence and the context in which they occur, and the perceived impacts of the violence on their well-being, health, and parenting. After the interviews, the participants completed a questionnaire to provide complementary sociodemographic information.

The interviews were conducted by graduate students, who received additional training from the community-based partners on data collection issues with female victims. The interviewers practiced role-playing to prepare them to respond to different scenarios. To ensure the participants’ safety, a security protocol was included in the training. In addition, they received ongoing supervision in the form of feedback on their interview. Furthermore, given the expected impact of repeated exposure to stories of violence, they could be debriefed at any time during the data collection.

### Data Analysis

With the participants’ agreement, all interviews were transcribed in their entirety, anonymized, and subjected to coding with NVivo12. A summary and graphic representation were also developed for each trajectory. The interview content was then thematically analyzed (Braun & Clarke, 2012). Next, through the use of matrices, the data were subjected to horizontal analysis to compare the stories in terms of similarities and differences and their divergences from different axes (Miles et al., 2013). Several methods were used to confirm the validity, credibility, and transferability of the results, including reflective note-taking by the research team members, an interjudge agreement procedure among the co-researchers and partners that supports the analysis strategies, and a discussion by the research team of the conceptual categories that emerged.

## Results

A descriptive sequential analysis of the DVPP experiences and the living conditions that undermined the women’s trajectories is presented, according to the participants’ perceptions. It shows the different forms in which the DV manifested in three periods: before the pregnancy, during the pregnancy, and after pregnancy, up until the child is 2 years old. The thematic analysis reveals the key events in these trajectories and sheds light on the various contexts that contributed to modulate the different forms of DVPP.

### Diverse Forms of Violence and Vulnerability Contexts Were Interwoven in the Women’s Trajectories

The participants recalled a variety of DVPP experiences (Table 2). They are grouped into seven categories: psychological, verbal, physical, control, financial, sexual, and reproductive coercion. Examples from the interviews are provided as illustrations. A single act could be interpreted simultaneously as more than one form of DVPP in the space of a given episode. The objective of this table is not to draw a quantitative portrait of DVPP, but instead to indicate the range and co-occurrence of the different forms of violence that were inflicted on the women at different times during this period.

**Table 2.** Co-Occurring Forms of DV Reported by Participants.

Forms of DV	Examples of manifestations of DV	Before pregnancy (*n =* 15)	During pregnancy (*n =* 17)	After childbirth (*n =* 16)
Psychological	Threats, intimidation, belittling—most often in connection with motherhood	12	16	16
Verbal	Yelling, insults—most often in connection with her physical appearance or parenting skills	10	15	13
Physical	Blows, strangulation, punching holes in walls, throwing or breaking objects	11	12	13
Control	Forbidding or restricting her movements, monitoring her activities, geo-tracking	8	8	13
Financial	Requiring her to pay his debts and expenses, theft	7	7	10
Sexual	Pressure or coercion through nonconsensual sexual relations	5	5	5
Reproductive coercion	Threats to separate if she terminates the pregnancy, threats or attempts to provoke a miscarriage, impeding her from accessing services and information about termination or continuance of the pregnancy	—	5	—
Co-occurring forms of violence		Before pregnancy (*n =* 15)^a^	During pregnancy (*n =* 17)	After childbirth (*n =* 16)
1 form		1	1	2
2–4 forms		8	8	5
5 or more forms		6	8	9

*Note. n* = 17. DV = domestic violence.

a Although the forms of violence and the co-occurrences in the different periods may refer to the same number of participants, they are not necessarily the same participants.

Almost all the participants (*n* = 15) reported at least one DV episode before their pregnancy, and all experienced DV during pregnancy. Except for one participant whose child was 2 months old at the time of the interview, all participants experienced DV after the baby was born. The most often mentioned form was psychological violence, for all three parts of the reference period. Women report that verbal violence was used more widely as a strategy to denigrate them during their pregnancy than it was before that pregnancy. They also report an increase in coercive control after childbirth. Women report that they suffer more forms of violence after childbirth, except for sexual violence, which showed similar frequency before and after the perinatal period: “I felt very pressured [. . .] I would do it with him, you know. I would say yes, but I felt pressured, as if I had no choice” (M6). For almost all the participants (*n* = 15), the different forms of violence were interwoven over the perinatal period.

The participants described two contexts that they felt made them more vulnerable and aggravated the violence, while normalizing it. Notably, they identified financial precarity and their partner’s abuse of alcohol and drugs. About two thirds of the participants (*n* = 12) had supported their partner financially, and subsequently the family. They explained this in different ways: The woman held a job and her partner did not, her job was better paid, or her partner refused to contribute equally to the expenses. For example, “He wasn’t working! So it was stressful, because we had to start buying things for the baby. [. . .] Honestly, there were many times when we couldn’t pay our bills, because he went out to buy pot or other drugs” (M12). However, two women were financially dependent on their partner:

I was also going through a hard time. I was vulnerable, big time! And he took advantage of that. He told me what I wanted to hear, and I fell right into his trap, [. . .] because I was going through a really hard time. It was like there was no way out. I was trying my best to get by, but I felt like I was stuck. (M10)

In many cases, the women’s precarious situation appeared to influence them to remain in the relationship; they were afraid they would be really poor if they ended it and became a single mother. In other cases, the women maintained the relationship because of their partner’s financial precarity, which they felt only exacerbated the violence.

Several participants (*n* = 9) said that the partner’s alcohol and/or drug abuse fueled his violent behavior. They described how it had begun with the pregnancy and then increased markedly over the perinatal period:

He drank a lot. When it’s just beer, that’s OK. But when he started on the rum first thing in the morning, like what happened later after we had the baby, at the end [. . .] I was scared. (M5)

The partner’s substance abuse created tension and was often accompanied by violent episodes. For example, a partner under the influence would be more aggressive or prone to use physical, economic, or psychological violence to force the woman to give him money or items to sell so he could buy more alcohol or drugs.

Two additional contexts appeared to contribute to the participants’ vulnerability: the residential situation and the immigration experience. The residential situation contributed somewhat to the women’s vulnerability. Some participants had moved in with their partner rapidly, as early as a few weeks after the first encounter. In one case, a woman who was pregnant from another man was at risk for homelessness when she met the partner who would perpetrate the violence. She moved in with him hastily because she was in a situation of insecurity and precarity, even though the violence had already begun by that time. Three participants were not living with their partner before or during the pregnancy, and the partner of one moved in right after the baby was born. Four participants associated moving in together with the emergence or increased frequency of violent behaviors, greater isolation from a support network, and more control over their day-to-day activities. One participant recounted how she had to move 4 times when the neighbors complained about her partner’s behavior: “It got harder and harder, because you’re moving all the time, [. . .] people ask questions, but me, well, I’m always trying to make excuses for him” (M12).

Many participants reported that these moves, sometimes one right after another, and especially from the city to a rural area, made them even more vulnerable:

So me, I wasn’t in the city anymore, [. . .] he was the one with the car. Me, I was all alone at home. I only got the car when I had an appointment. So, out in the middle of nowhere without a car, you don’t get around much. I was shut in. I felt [. . .] even more so in that house, [. . .] and it ended up being a jail. (M13)

To the lack of mobility that accentuated their solitude, we may add increasingly violent behaviors by the partner, according to many of the participants:

He was even more aggressive because I was even more isolated. [. . .] We had even fewer neighbors nearby, so I no longer had a circle of friends. [. . .] I didn’t do anything anymore because I was always cooped up at home. (M5)

Several participants were born outside Canada (*n* = 7). Some had immigrated to Canada with their family when they were children, and others had come alone as adults. Still, others had come with their partner. Without being directly questioned about it, three women identified how their migratory situation (living in another country than the rest of their larger family) may have influenced the situation of DV by contributing to their further vulnerability and isolation. One participant disclosed that the stress of settling and integrating into the new country could have accentuated some conflicts with her partner, but she clearly distinguished between that earlier time and the time when the DV began. She described the cultural shock they had faced as a couple. It is important to mention that all the immigrant participants had obtained regular status: They were neither in an irregular immigration situation nor an asylum seeker. Their ties with at least two countries meant travels that the partner could use for purposes of control. Thus, in two cases, during a trip to the home country, the partner confiscated the mother’s and children’s passports so he could control their movements:

Me, I travelled to [country of origin] for a vacation. [. . .] On the tenth day, he told me, “[. . .] I’m going back to Canada, but you, you’re going to stay in [country of origin] and I only want my children [. . .] to return to Canada.” (M9)

Other participants said that their partner had contacted their family back home to pressure them, influence their decisions, or damage their reputation. For example, one participant reports that after several episodes of sexual violence, she became pregnant. Her partner then announced the pregnancy to her family back home, without her consent and while she was still ambivalent about whether or not to pursue the pregnancy. Her partner threatened to sue her if she had an abortion, even though this is not a possibility in Canada. She also reports that in her parent’s culture, abortion is very frowned upon and still considered a crime. Because her partner kept her from seeing her friends, she reports feeling alone and lost at that time. Under pressure, she pursued the pregnancy.

### From the Announcement of the Pregnancy to Childbirth: The Diverse Faces of Violence

The 17 participants accounted for a total of 26 full-term or ongoing pregnancies in relationships marked by DV in the last 5 years. Of these pregnancies, only 10 were planned, whereas 16 were unplanned. The announcement of the pregnancy sometimes was an opportunity for the partner to use violence in a new way, such as casting doubt on the child’s paternity and accusing the mother of infidelity (*n* = 3). In two cases, the father’s insistence on choosing the baby’s first name was the catalyst for violence.

Five participants experienced reproductive coercion. Their partner had either threatened to provoke a miscarriage or attempted to do so by hitting her in the stomach, slamming her up against a wall, or poisoning her: “He even tried to make me drink bleach in hopes that I would miscarry” (M1). Other partners had threatened to break up unless the woman had an abortion. Inversely, even though they preferred an abortion, some participants realized that they had no choice but to accept the pregnancy, or at least they needed more time to consider their options. In the majority of cases, the partners used pressure, threats, physical violence, or economic restrictions in their attempts to prevent the women from accessing services: “But he told me, ‘If you get an abortion, I’m going to make you regret it. [. . .]. I’m going to report you’” (M10). In cases where the women were pressured to get pregnant or continue a pregnancy, the partners did not seem to be involved in either the pregnancy follow-ups or childcare.

Many participants reported that their partner did not do his share of routine household tasks during the pregnancy. Many said that their partners were also inconsiderate about the pregnancy itself: “He didn’t do any shopping. [. . .] He didn’t come to my appointments. I went by myself. [. . .] He didn’t want to know anything about it” (M1). This participant and others added that her partner found the idea of having a child repellent. Many participants had hoped that the violence would stop when they became pregnant, or that at least it would decrease, and that their partner would be more interested in supporting them. However, all the participants had experienced at least one violent episode during their pregnancy. Many of them identified the pregnancy as a turning point in the DV dynamic. For example, one participant said that her partner “got meaner,” and in addition to unfairly accusing her of being unfaithful, he disparaged her body and her character.

In some cases, new forms of violence appeared during the pregnancy: “And then, after that, it really began: serious punches, all over my body. It was raining blows. [. . .] Every time we had a misunderstanding, he reacted by hitting me” (M10). Another participant reported that during her third pregnancy, her partner began secretly recording her at home when he was out, using a concealed microphone, which as far as she knew was a new behavior. Other participants told us about financial violence that targeted everything to do with the pregnancy and the preparations for the baby’s arrival: “You know, he wanted to stop me from putting any money aside for the baby” (M1).

The majority of the participants (*n* = 12) felt that the violence increased during their pregnancy: “The violence got ten times worse. [. . .] It was horrible. [. . .] He became another person. [. . .] He was hateful! I was terrified” (M1). Most participants said that the different forms of violence (physical, psychological, or verbal) got worse during the pregnancy compared to before. Five participants felt that the situation had deteriorated progressively, going from bad to worse with each pregnancy. Other participants said that the violence had subsided, but that there were still some episodes. One participant told us that her partner modified his manner of physical violence once her stomach got bigger:

So that was when he, like [. . .] the stomach. I was showing, so maybe that was when he let up a little! But not really, either, you know, because he kept on giving me slaps, you know. [. . .] Or he did things that according to him wouldn’t hurt the baby, that kind of thing, you know! Pulling my hair, slapping me, pushing me around, but you know, not the stomach. (M12)

Other participants also recalled some calm intervals during their pregnancy: “He spent less time sulking. [. . .] He was faster to act sweet again after his angry episodes” (M3). Other partners apologized after violent episodes: “He became like, [. . .] he fell into a rage, a very black rage, and afterwards he was nice again, like an angel. [. . .] He asked me to forgive him, said he was sorry” (M10). Nevertheless, after the honeymoon was over, the violence resurfaced:

Because every time I came home [. . .] he put me down. He insulted me all the time. And afterwards, it was like a honeymoon all over again. And he was super affectionate, super correct. But at that time, he wanted to change. [. . .] But in the end, a few months, two months later, he was back to square one. (M4)

The most often reported form of violence during this part of the perinatal period was psychological (*n* = 16). In some cases, it was directly related to the pregnancy, for example, when the partner prevented her from resting or sleeping: “When I went to bed, he came into the bedroom, turned on the light, and pulled off the bed covers” (M11). The psychological violence occurred partly in relation to the inherent changes during pregnancy, in the sense that the partner could use specific arguments that did not apply in other circumstances. For example, he might complain that her weight gain made the mother unattractive, threaten to take the newborn baby away from her, or blame her for placing the baby’s health at risk:

Then my partner, after that, he started telling me all the time that if I had heartburn, because I was constantly throwing up, and I didn’t feel very good, and I had a headache, and I was tired, [. . .] it was because, [. . .] it was because, [. . .] I didn’t love my baby. [. . .] And, I was going to make it sick; I was going to make it handicapped. (M7)

In addition, several women report they were regularly afraid of their partner: “I was so stressed out, so distressed! Every time I saw him, it was like: I had a lump in my stomach” (M10). Many have gradually developed a certain vigilance in anticipating a new episode of violence: “It felt as if I was walking on eggshells, trying not to displease him so he wouldn’t burst” (M11).

Some partners threatened to harm the fetus directly. For example, one partner insinuated that he could provoke a miscarriage: “If you fall asleep, well, you might just lose something. [. . .] It might just happen that when you’re taking a walk, when you’re going down the stairs, that you’ll take a fall. One kick and it’ll be gone” (M1). A few women received personal death threats during their pregnancy. One participant who had decided to break up with her partner was explicitly threatened: “Well, because he told me that he was going to kill me, I didn’t dare go see him in person in case he carried out his threat” (M1). Two women found out that their partner had a gun.

According to many of their stories, the participants were also exposed to violent behaviors during the childbirth. Thus, some (*n* = 4) recalled DV episodes when they were in labor. The forms of violence included psychological (death threats, refusal to accompany her to the hospital, belittling and mocking her during childbirth, taking photos during childbirth without her consent and posting them on social media), physical (*n* = 1; strangulation), and verbal (insults). One participant was blamed by her partner for exaggerating the pain of her contractions while she waited for the taxi to take her to the hospital: “Then he yelled at me, oh, in the street, and he said, ‘Oh, you’re just a piece of shit, you’re just this, you’re just that, [. . .] You’re always seeking attention! [. . .] You’ve got a bitchy attitude!’” (M12). Another participant felt that her partner enjoyed seeing her suffer during the childbirth. One participant described how, in the midst of her worst contractions, her partner started to threaten her and act aggressively: “I was in the middle of giving birth. I was having contractions, but I had to run around the table while I was contracting because he was trying to strangle me” (M5). Two participants asked their partner, in vain, that he not be present for the childbirth because they were afraid that it would only add to their stress. In another case, the staff had to implement security measures to prevent the partner from entering the birthing room.

### The Postnatal Period: Increased Responsibility for Women, and Increased Violence by Their Partners

Almost all participants experienced at least one DV episode in the postnatal period. Only one said that the violence stopped after the birth of her baby, who was 2 months old at the time of the interview. For the majority (*n* = 14), this was a time of repeated victimization and more frequent violent episodes that increased in severity: “That’s when he really started attacking me, there, straight out, after my daughter was born” (M17). Another participant said that the violence and crises got worse by the day: “Every day there were mini crises, but, [. . .] I’d say that, once every two weeks there were major crises that ended with me being slammed up against the wall!” (M12). The DV continued to build up and got increasingly worse as the children grew up: “The violence escalated over the years” (M3). In addition, even though two participants broke up with their partner while they were pregnant, the partner continued to harass them with cyberbullying for several months after the baby was born: “He would be sending me messages like ‘I know where you are! [. . .] If I can’t have you, nobody will. You’ll see’” (M1).

Some mothers had babies who were premature, hospitalized, or ill. These participants went alone to the medical follow-ups and physician consultations. Thus, they assumed sole responsibility for understanding sometimes complicated medical situations, which they then had to explain to their partner. The partner would then seize the opportunity to discredit or question her parenting skills and her ability to retain information. Similarly, one participant had felt completely alone since the first days following a premature birth. She realized that she could not count on her partner: “He never came once to the hospital. [. . .] I was all by myself” (M11).

Other participants reported that their partner exercised less control over them during the first few weeks after childbirth. Their explanation was that the partner knew that she was at home taking care of the newborn, and therefore did not ask her to account for herself as much:

When I gave birth, it was during a heat wave, [. . .] so there was no way I could go out. So, seeing that I was always with my daughter, and when he wasn’t there, well, he knew that I wasn’t going out cruising or hanging out in bars, or whatever, going to see my friends. (M13)

Nevertheless, after the first few weeks, the controlling behaviors reappeared and got worse.

Most of the participants felt that they were solely in charge of the childcare, and that their partners were not very or not at all invested. Nor did their partners do their share of the housework after the baby was born. Some fathers refused to assume their parenting responsibilities, or even acknowledge that they were the father. According to one participant, “Before we separated, his favorite subject was that his son, [. . .] ‘the baby was not his son’” (M12). Not only were the majority of the participants solely responsible for their child’s well-being, but many said that their partner prevented them from sleeping, resting, or going to their family for support. This made them even more tired and contributed to the violence-related stress. To this was added the father’s lack of investment in financial and family planning (*n =* 7), making the mother’s mental burden that much heavier. Many participants felt that they never got a break because they were constantly responsible for the baby and were made to feel guilty if they took any time for themselves: “When I took a shower, he’d be standing in the doorway holding the baby. And then he’d make the baby cry. [. . .] He’d stand there and tell me, ‘Well, hurry up!’ [. . .] I never got a break” (M8).

Similarly, several participants reported that when they were out and the partner was supposed to take care of the baby, he would badger her on the phone. Sometimes, this control included checking up on who she was seeing, which limited her interactions and increased her feelings of isolation:

Other times, he would call me non-stop. I might be at the park with my daughter, and he would call me over, and over, and over. Just to make sure that I wasn’t talking with some man in the park, so I wouldn’t talk with anybody there. (M13)

On the contrary, many participants felt that the violence was a reaction to the attention she gave to the baby. In these cases, their partner’s behavior could be explained in part by his jealousy of all the attention and care she gave to her newborn or infant: “Once I had established a [. . .] closeness with the baby. [. . .] It made him really jealous. Then, sort of defensive. Then, frustrated” (M3). Others blamed themselves for not cooking or doing the housework as much as before, and being too involved in mothering tasks:

And the time that it happened, me, I was lying down with the baby, breastfeeding. “Leave the baby alone now. Start cooking! The baby’s not a doll,” and that kind of stuff. “Since he came, it’s like you don’t care anymore about [. . .].” He insulted me in front of everybody, put me down, and like that. (M10)

Furthermore, besides being solely responsible for the childcare, the great majority of the participants continued to experience different forms of violence. These manifested as belittlement of her mothering role, her relationship with the baby, and her ability to carry out her responsibilities.

Overall, the partners were not invested in fatherhood. Nevertheless, many of them used the baby’s presence to intensify their control over and violence against the mother. For example, four participants recounted that their partner had tried to hit them or to punish them by restricting their access to the baby and preventing them from holding it. Other participants had been subjected to physical violence when they were holding the baby, or verbal violence in the baby’s presence: “He pushed me. [. . .] Y’know, sometimes I’d be holding the baby, like this, and we’re talking about a three-and-a-half-month-old, maybe four months, and [. . .] he’d throw me up against the wall” (M12).

Some partners threatened to harm the baby or even kill it. One participant heard her partner say, more than once, “I’m going to break his neck. [. . .] I’m going to kill you and your damn baby” (M5). Further along in the postnatal period, the participants became more concerned about the consequences of exposing their baby to violence and threats:

So then my son, at that time, he would either freeze or burst out crying, and go “Maaa!!!” He was witnessing more and more times when my ex, he would back us into a corner, and then yell at me. (M8)

Moreover, although the physical violence was usually directed toward the participants, sometimes the child was on the receiving end (*n* = 7): “When my child was about two, he started in fact, to . . . some violence towards my daughter. [. . .] And she would be crying. She would say: Mommy, boo boo” (M4). The violent behaviors toward children included arm twisting, face slapping, shaking, breaking ribs, ripping up clothes, and wrecking the car seat. In such contexts, many participants feared for their child’s safety and development.

Finally, a majority of participants report that the DV aimed to lower their self-esteem and degrade their image as a mother by repeatedly questioning their skills or their choice as a parent: “I would always question my choices as a mother” (M9). Several report having suffered verbal and psychological violence in this regard. Others say their partner made them feel guilty whenever they were away from home, even for a short time, sending them back to the image of being a bad mother: “During the time I was away from the apartment, he would be calling me non-stop. He would put my son on the line and make him cry” (M8). Many also believe that being hit and insulted in front of their child has also actively contributed to reduced self-confidence and increased shame, as one mother reports as follows: “I did not have a lot of confidence in myself to begin with and then he . . . he crushed it again . . . at its lower” (M5).

## Discussion

The main objective of this article was to document the manifestations of DV that occur during pregnancy and the 2 years following childbirth. Mothers who had experienced DV during the perinatal period were interviewed and encouraged to share their stories. Overall, the results corroborate prior findings (Edin et al., 2010; James et al., 2013; Vatnar & Bjorkly, 2010) that pregnancy is rarely a time of respite from DV, despite the hopes of many women. More specifically, the participants’ stories told how the violence can grow worse during pregnancy, from the first announcement of the pregnancy up to childbirth, which itself sets the stage for diverse acts of violence. All the participants experienced violence when they were pregnant. While the most often reported forms of violence were psychological and verbal violence in connection with parenting skills and perceptions, the participants also reported the presence of simultaneous forms of violence. In addition, control by the partner acted to limit their freedom of movement and action, isolate them from their social circle, and deprive them of financial autonomy. Moreover, several participants said that they had received death threats when they were pregnant, concurring with previous research (Edin et al., 2010).

Most importantly, the narratives of the women we met highlight that violence was part of their daily lives during the perinatal period, in one form or another. Their experience is part of a dynamic of violence and control, which goes beyond the manifestation of a specific act of violence or the presence of a single form. It is therefore this reality of intertwined violence that our data illustrate. This understanding of the co-occurrence of forms of violence is important to document and measure, as it reveals a context of severe victimization, as depicted in other qualitative studies focusing on violence and the perinatal period (Buchanan & Humphreys, 2021; Edin et al., 2010). This reality seems less described by studies that have quantitatively taken an interest in this phenomenon; these often measure a limited number of forms of violence or dichotomize violence according to its absence or presence (Daoud et al., 2012; James et al., 2013; Taillieu & Brownridge, 2010). However, these means of measuring or reporting the prevalence of violence may underestimate the magnitude of the problem or mask the specifics of some experiences. The presence of measurement tools that integrate the contexts of coercive violence during the perinatal period would be a promising avenue. Moreover, the different forms of violence that were revealed throughout the interviews raise questions about how cases of DV are generally identified in clinical settings. The usual indicators of potential DV have been certain signs or characteristics. However, certain forms of violence could be harder to detect. Some authors posit that the difficulty of documenting sexual violence could be due to the taboos that surround it (Vatnar & Bjorkly, 2010). Case identification strategies should therefore include questions that encourage women to talk about violent behaviors in a wider sense, besides physical violence.

Another result worthy of attention is the high number of unplanned pregnancies. Of the 26 pregnancies covered in this study, more than half were unplanned. This is a worrisome issue that merits greater attention, particularly given the consequences for the health, well-being, and socioprofessional and academic trajectories of women who are obliged to continue a pregnancy when they would prefer to terminate it (Biggs et al., 2017; Foster et al., 2015, 2018). For instance, compared with women who were able to terminate an unwanted pregnancy, those who did not have that choice were more likely to be poor, less likely to hold a full-time job, and more likely to receive social assistance (Foster et al., 2018). Therefore, more systematic DV detection and tracking could be integrated into pregnancy consultations. For example, health care professionals could ask pregnant women, when they are alone, whether the pregnancy was wanted or planned, and they could provide information on alternatives if requested. Interdisciplinary collaboration with other social actors could broaden the support offer and improve the effectiveness of interventions (Shorey et al., 2014).

However, while case identification during pregnancy is important for providing appropriate support and interventions for pregnant women who are victims of violence by their intimate partner (ACOG Committee for Health Care on Underserved Women, 2012; National Institute for Health and Care Excellence [NICE], 2014; U.S. Preventive Services Task Force, 2019; WHO, 2013), our results indicate that preventive measures should also target women who are not yet pregnant. In our sample, the DV preceded the pregnancy in 15 out of 17 cases, echoing the findings of James et al.’s (2013) meta-analysis, which reveals a fourfold higher risk of DVPP in the presence of a history of violence. Moreover, our results confirm the relevance of interventions that target early childhood, when this social and health issue is also present. For 16 of our 17 participants, the DVPP continued after childbirth, with increasing intensity and in diversified forms. As it happens, the postnatal period is a time of regular health care consultations for vaccinations, follow-up on the baby’s health and breastfeeding, and so on (Laforest, Gamache, & Poissant, 2018). This provides an opportune time to reduce DVPP through awareness raising among health care providers and the use of interdisciplinary protocols to identify problems and support families.

The novelty of our study lies in the consideration of violence during childbirth. Although reported by only a few participants, the presence of violence during labor and childbirth is alarming. The women who were affected did not give birth in ideal conditions, among other reasons because they were under considerable stress and did not have access to a supportive birthing companion. The literature clearly shows that continuous support during childbirth is a key factor for optimal physiological birth without intervention (Institut national d’excellence en santé et en services sociaux [INESSS], 2012). In light of the participants’ stories, the presence of a violent partner may be considered a threat to the birthing process. It would be relevant therefore to raise awareness among obstetric care professionals and provide them with training in the various possible manifestations of DV, including the different forms of control and psychological and verbal violence. A key element in awareness raising would be to systematically ascertain, when the woman is alone, whether or not she wants her partner present during childbirth, and to respect her decision even if her partner insists. If a woman refuses to have her partner present, it would be pertinent, to prevent possible consequences once she goes back home, to offer her accommodation at a secure shelter when she leaves the hospital, if she wishes, and to provide her with a list of useful resources. In addition, care protocols and references could be provided in the birthing units, in collaboration with the hospital’s social workers and psychologists and community groups working with violence issues.

One of the significant findings of this study concerns the partner’s lack of investment in parenting and the over-responsibilization of women. Many of the participants recalled that they had to take care of the baby and the household tasks by themselves. This heavy responsibility and the associated mental burden, combined with contexts of vulnerability (partner’s substance abuse, financial precariousness), made it harder for them to fully exercise their parenting role and to derive enjoyment and satisfaction from it. Such contexts also impede children from developing and bonding with their parents (Buchanan et al., 2014; Katz, 2015; Levendosky et al., 2011).

### Limitations

This study includes certain limitations that limit the transferability and interpretation of the results. First, the women recounted their experiences of victimization by responding to semi-directed interview questions and depending on whether they were ready to share their stories. Consequently, the itemization and classification (Table 2) of the forms of violence and their occurrence are necessarily incomplete and imprecise because the classification is based on our analysis grid. In addition, social desirability and memory bias, due to the self-reference effect over the 5 years considered in this study, could have colored their stories and overshadowed some events. Finally, the small sample size does not allow identifying the diversity of trajectories needed to determine relationships between the mother’s social position and the DVPP experience. For example, while we were able to meet a number of women born outside of Canada who explained how their migration status influenced their experience of DV, we did not question racialization as an oppressive system and how that might also influence their trajectory. We also did not question whether or not the participants lived with a disability and, if so, how this could have affected their history. In addition, heterosexuality was not an inclusion criterion, but we did not meet any women declaring another sexual orientation. In light of these limitations, our recruitment tools could undoubtedly have been more comprehensive on the inclusion of these situations. Consequently, despite the care taken to recruit participants with different profiles, the empirical corpus does not enable obtaining data saturation in this respect, on one hand, because of a small number of participants and, on the other hand, because we may not have delved into the particularity of certain experiences such as those of racialized women.

### Implications for Future Research

Many potential research avenues emerge from this study. First, it would be informative to obtain longitudinal qualitative data on DV, particularly covering the perinatal period and the child’s first years of life. This would involve, for example, following a cohort of women by interviewing them every 6 months over a 5-year period regarding the presence of DVPP and their experience of parenthood (with a newborn, a baby, or a toddler). This would enable a more substantial understanding of the life trajectory in terms of the violence experienced during parenthood. Of course, the goal would not be to compare one group of women receiving support for IPV with another group not receiving it. At each data collection period, individuals would receive a brochure featuring various DV services. The use or not of these services would be a component of the study. Furthermore, it would be useful to ensure that all relevant voices are heard to broaden our understanding of the experiences. For instance, our results indicate that the domestic situation and the immigration context can pose specific challenges. Future studies are needed to give women in these circumstances opportunities to speak. In addition, we need to hear from women who were pressured to terminate their pregnancy and women whose pregnancies were unplanned. Sexual and reproductive health, including childbirth and breastfeeding, have been underrepresented in studies on DV and motherhood, despite their central place in women’s lives. In the future, research designs and methods could also integrate intersectional perspectives to better document the experiences of more marginalized women and enrich our comprehension of the different ways that oppression influences the victimization experience in DVPP contexts.
